# Multi-channel EEG recordings during a sustained-attention driving task

**DOI:** 10.1038/s41597-019-0027-4

**Published:** 2019-04-05

**Authors:** Zehong Cao, Chun-Hsiang Chuang, Jung-Kai King, Chin-Teng Lin

**Affiliations:** 1 Discipline of ICT, School of Technology, Environments and Design, College of Sciences and Engineering, University of Tasmania, Hobart, TAS Australia; 2 Department of Computer Science and Engineering, National Taiwan Ocean University, Keelung, Taiwan; 3 Brain Research Center, National Chiao Tung University, Hsinchu, Taiwan; 4 Centre for Artificial Intelligence, Faculty of Engineering and IT, University of Technology Sydney, Sydney, NSW Australia

**Keywords:** Attention, Electroencephalography - EEG

## Abstract

We describe driver behaviour and brain dynamics acquired from a 90-minute sustained-attention task in an immersive driving simulator. The data included 62 sessions of 32-channel electroencephalography (EEG) data for 27 subjects driving on a four-lane highway who were instructed to keep the car cruising in the centre of the lane. Lane-departure events were randomly induced to cause the car to drift from the original cruising lane towards the left or right lane. A complete trial included events with deviation onset, response onset, and response offset. The next trial, in which the subject was instructed to drive back to the original cruising lane, began 5–10 seconds after finishing the previous trial. We believe that this dataset will lead to the development of novel neural processing methodology that can be used to index brain cortical dynamics and detect driving fatigue and drowsiness. This publicly available dataset will be beneficial to the neuroscience and brain-computer interface communities.

## Abstract (summary)

**Table:** 

Design Type(s)	behavioral data analysis objective • source-based data analysis objective • stimulus or stress design
Measurement Type(s)	brain activity measurement
Technology Type(s)	electroencephalography
Factor Type(s)	
Sample Characteristic(s)	Homo sapiens • brain

Machine-accessible metadata file describing the reported data (ISA-Tab format)

## Background & Summary

Driving safety has attracted public attention due to the increasing number of road traffic accidents. Risky driving states, such as fatigue and drowsiness, increase drivers’ risk of crashing, as fatigue suppresses driver performance, including awareness, recognition and directional control of the car^1^. In particular, high levels of fatigue and drowsiness diminish driver arousal and information processing abilities in unusual and emergency situations^2^.

During a sustained-attention driving task, fatigue and drowsiness are reflected in driver behaviours and brain dynamics^3^. Furthermore, electroencephalography (EEG) is the preferred method for human brain electrophysiological monitoring while performing tasks involving natural movements in a real-world environment^4^. In 2003, we began conducting laboratory-based experiments collecting EEG data to investigate brain function associated with sustained attention during a safe driving task^5,6^. Our experiments have two distinct goals: (1) evaluating neurocognitive performance, i.e., determining key signatures of how the neurocognitive state of the driver (e.g., physical and physiological) varies when faced with the sensory, perceptual and cognitive demands of a sustained-attention situation^7–10^; and (2) developing advanced computational approaches, i.e., investigating novel computational, statistical modelling and data visualisation techniques to extract signatures of neurocognitive performance, including novel analytic and algorithmic approaches for individually assessing drivers’ neurocognitive state and performance^11–13^.

To acquire the experimental dataset, we adopted an event-related lane-departure paradigm in a virtual-reality (VR) dynamic driving simulator to quantitatively measure brain EEG dynamics along with fluctuations in behavioural performance. All of the participants were required to have a driver’s licence, and none of them had a history of psychological disorders. The 32-channel EEG signals and vehicle position were recorded simultaneously, and all of the participants were instructed to sustain their attention in this driving experiment.

Several research studies on driving performance, including kinaesthetic effects, mind-wandering trends and the development of drowsiness prediction systems, have been conducted by our team using this EEG dataset. Specifically, to study EEG dynamics in response to kinaesthetic stimuli during driving, we used a VR-based driving simulator with a motion platform to produce a somatic sensation similar to real-world situations^14^. For mind-wandering trends, we investigated brain dynamics and behavioural changes in individuals experiencing low perceptual demands during a sustained-attention task^15^. In terms of the drowsiness prediction system, we proposed a brain-computer interface-based approach using spectral dynamics to classify driver alertness and predict response times^16–20^. We determined the amount of cognitive state information that can be extracted from noninvasively recorded EEG data and the feasibility of online assessment and rectification of brain networks exhibiting characteristic dynamic patterns in response to cognitive challenges.

These data descriptors describe a large EEG dataset in a sustained-attention driving task. We aim to help researchers reuse this dataset to further study the behavioural decision making of drivers under stress and cognitive fatigue in complex operational environments, such as car driving with kinaesthetic stimuli, which requires directly studying the interactions among the brain, behaviour, the sensory system and performance dynamics based on simultaneous measurements and joint analysis. We expect that this dataset could be used to explore principles and methods for the design of individualised real-time neuroergonomic systems to enhance the situational awareness and decision making of drivers under several forms of stress and cognitive fatigue, thereby improving total human-system performance. We believe this research will benefit the neuroscience and brain-computer interface communities.

## Methods

### Participants

Twenty-seven voluntary participants (age: 22–28 years) who were students or staff of the National Chiao Tung University were recruited to participate in a 90-minute sustained-attention driving task at multiple times on the same or different days. In total, 62 EEG data sets were collected from these participants. The participants had normal or corrected-to-normal vision. In addition, none of the participants reported sleep deprivation in the preceding weeks, and none had a history of drug abuse according to the self-report. Every participant was required to have a normal work and rest cycle, get enough sleep (approximately 8 h of sleep each night) and not stay up late (no later than 11:00 PM) for a week before the experiment. Additionally, the participants did not imbibe alcohol or caffeinated drinks or participate in strenuous exercise a day before the experiments. At the beginning of the experiment, a pre-test session was conducted to ensure the participants understood the instructions and to confirm that none were affected by simulator-induced nausea. This study was performed in strict accordance with the recommendations in the Guide for the Committee of Laboratory Care and Use of the National Chiao Tung University, Taiwan. The Institutional Review Board of the Veterans General Hospital, Taipei, Taiwan, approved the study. All of the participants were asked to read and sign an informed consent form before participating in the EEG experiments. The monetary compensation for one experimental session was approximately USD $20.

### Virtual-reality driving environment

A VR driving environment with a dynamic driving simulator mounted on a six-degree-of-freedom Stewart motion platform was built to mirror reality behind the wheel. Six interactive highway driving scenes synchronised over local area networks were projected onto the screens at viewing angles of 0°, 42°, 84°, 180°, 276° and 318° to provide a nearly complete 360° visual field. The dimensions of the six directional scenes were 300 × 225 (width × height) cm, 290 × 225 cm, 260 × 195 cm, 520 × 195 cm, 260 × 195 cm, and 290 × 225 cm, respectively.

**Fig. 1:**
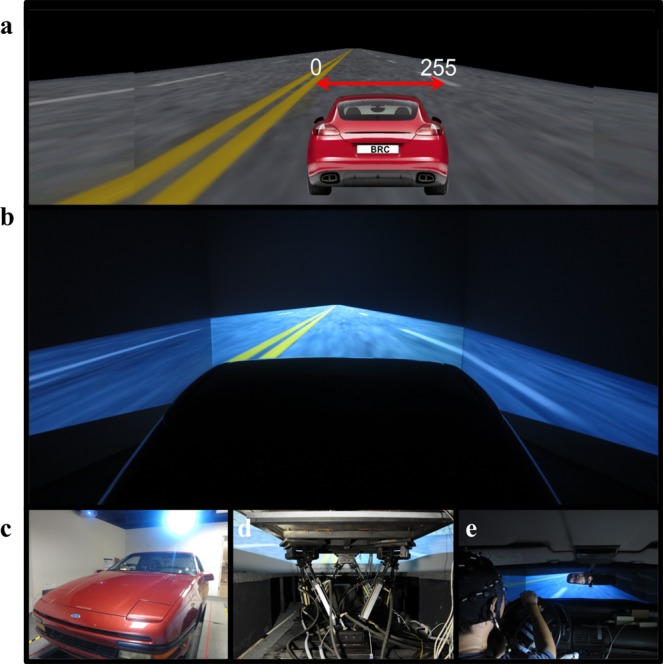
An event-related lane-departure paradigm in a virtual-reality (VR) dynamic driving simulator.

As shown in Fig. 1a,b, the experimental scenario involved a visually monotonous and unexciting night-time drive on a straight four-lane divided highway without other traffic. The distance from the left side to the right side of the road and the vehicle trajectory were quantised into values from 0–255, and the width of each lane was 60 units. The refresh rate of the scenario frame was set to emulate cruising at a speed of 100 km/hr. A real vehicle frame (Make: Ford; Model: Probe) (Fig. 1c) that included no unnecessary weight (such as an engine, wheels, and other components) was mounted on a six-degree-of-freedom Stewart motion platform (Fig. 1d). In addition, the driver’s view of the VR driving environment was recorded and is shown in Fig. 1e.

### Experimental paradigm

An event-related lane-departure paradigm^3^ was implemented in the VR-based driving simulator using WorldToolKit (WTK) R9 Direct and Visual C++. The paradigm was designed to quantitatively measure the subject’s reaction time to perturbations during a continuous driving task. The experimental paradigm simulated night-time driving on a four-lane highway, and the subject was asked to keep the car cruising in the centre of the lane. The simulation was designed to mimic non-ideal road surface that caused the car to drift with equal probability to the right or left of the third lane. The driving task continued for 90 minutes without breaks. Drivers’ activities were monitored from the scene control room via a surveillance video camera mounted on the dashboard. Lane-departure trials were obtained from experimental data collected from 2005 to 2012 at National Chiao Tung University, Taiwan.

**Fig. 2:**
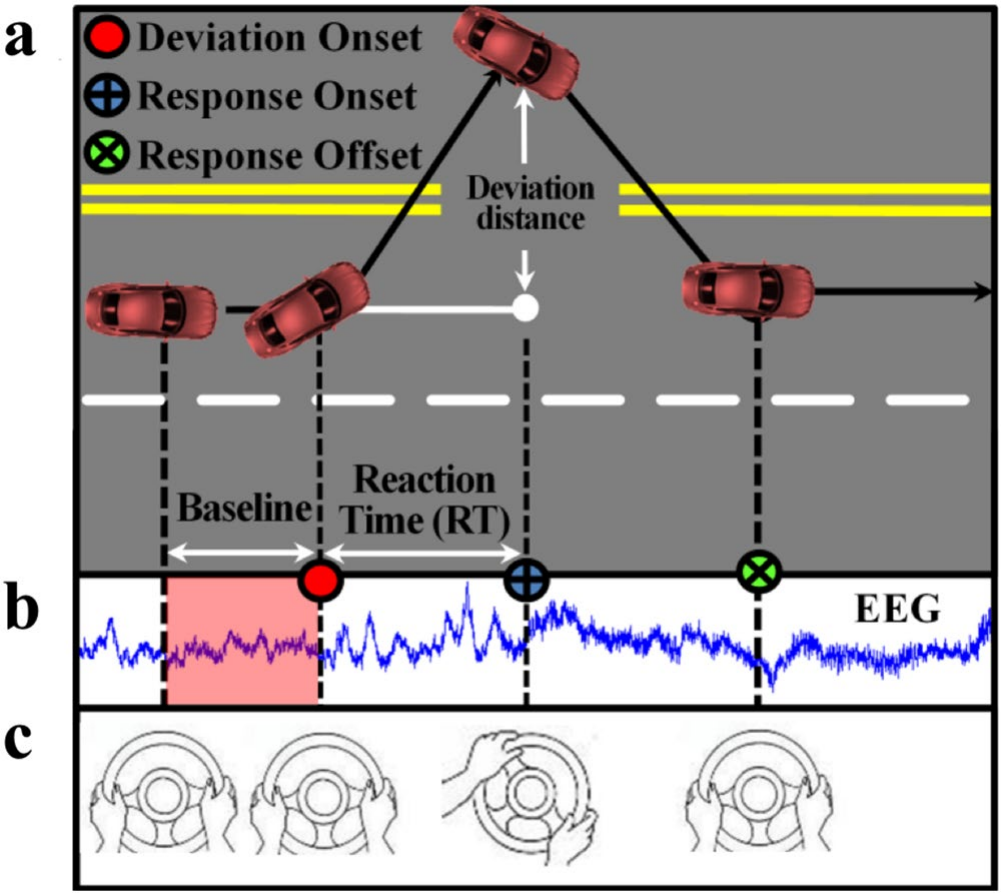
Experimental design. (**a**) Event-related lane-deviation paradigm. (**b**,**c**) EEG and behaviour were recorded simultaneously.

As shown in Fig. 2a, lane-departure events were randomly induced to make the car drift from the original cruising lane towards the left or right sides (deviation onset). Each participant was instructed to quickly compensate for this perturbation by steering the wheel (response onset) to cause the car move back to the original cruising lane (response offset). To avoid the impacts of other factors during the task, participants only reacted to the lane-perturbation event by turning the steering wheel and did not have to control the accelerator or brake pedals in this experiment. Each lane-departure event was defined as a “trial,” including a baseline period, deviation onset, response onset and response offset. EEG signals were recorded simultaneously (Fig. 2b). Additionally, the corresponding directions of turning the steering wheel are shown in Fig. 2c. Of note, the next trial occurred within a 5–10 second interval after finishing the current trial, during which the subject had to maneuverer the car back to the centre line of the third car lane. If the participant fell asleep during the experiment, no feedback was provided to alert him/her.

## Data Records

### Data recording and storage

During the experiment, the stimulus computer that generated the VR scene recoded the trajectories of the car and the events with time points in a “log” file. The stimulus computer also sent synchronised triggers (also recorded in the “log” file) to the Neuroscan EEG acquisition system. Concurrently, the Neuroscan system recoded EEG data with the time stamps of triggers in an “ev2” file. Because the number of time points in both recorded files was different, the first step was to integrate the two files into a new file with aligned event timing and behavioural data. The new event file was then imported by EEGLAB in MATLAB.

EEG signals were obtained using the Scan SynAmps2 Express system (Compumedics Ltd., VIC, Australia). Recorded EEG signals were collected using a wired EEG cap with 32 Ag/AgCl electrodes, including 30 EEG electrodes and 2 reference electrodes (opposite lateral mastoids). The EEG electrodes were placed according to a modified international 10–20 system. The contact impedance between all electrodes and the skin was kept under 5 kΩ. The EEG recordings were amplified by the Scan SynAmps2 Express system (Compumedics Ltd., VIC, Australia) and digitised at 500 Hz (resolution: 16 bits). Neuroscan’s Scan 4.5 is the ultimate tool for data acquisition. The acquired raw data were saved as .cnt files on the PC and server.

### EEG signals

**Fig. 3:**
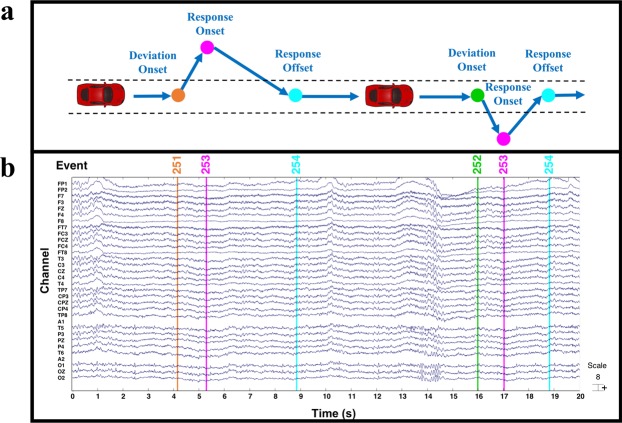
An example of behaviour and EEG performance. (**a**) Behaviour performance. (**b**) EEG signals with associated events.

The raw files were read using the EEGLAB toolbox in MATLAB. The uploaded files named with set suffixes contain all of the signals. After loading the files, the “EEG.data” variable included 32 EEG signals and one signal for vehicle position. The first 32 signals were from the Fp1, Fp2, F7, F3, Fz, F4, F8, FT7, FC3, FCZ, FC4, FT8, T3, C3, Cz, C4, T4, TP7, CP3, CPz, CP4, TP8, A1, T5, P3, PZ, P4, T6, A2, O1, Oz and O2 electrodes. Two electrodes (A1 and A2) were references placed on the mastoid bones. The 33rd signal was used to describe the position of the simulated vehicle. Additionally, as shown in Table 1, the types of events (see “EEG.event.type”) in the dataset were classified as deviation onset (mark: 251 or 252), response onset (mark 253) or response offset (mark 254). Of note, the time period between deviation onset and response onset was defined as reaction time (RT). Figure 3 shows an example of behavioural performance (Fig. 3a) and EEG signals (Fig. 3b) with associated events. Additionally, as shown in Table 2, we report the number of sessions per subject and include summary statistics on the number of events (including deviation onset, response onset, and response offset) per subject.

**Table 1:** Types of events in dataset.

EEG.event.type	251	252	253	254
Definition	Deviation onset (left)	Deviation onset (right)	Response onset	Response offset

**Table 2:** The numbers of sessions and events per subject.

Subject No.	Number of Sessions	Numbers of Events
S01	5	4827
S02	2	2028
S04	1	1083
S05	4	6378
S06	1	1077
S09	3	2112
S11	1	1290
S12	2	1869
S13	2	2244
S14	2	2181
S22	4	5022
S23	1	1317
S31	2	3618
S35	2	3285
S40	2	3921
S41	5	6747
S42	2	2430
S43	3	5709
S44	4	7269
S45	2	4023
S48	1	1050
S49	3	3102
S50	2	2085
S52	1	717
S53	3	3654
S54	1	615
S55	1	1923
**Total**	**62**	**81576**

Of note, we have uploaded the raw experimental dataset^21^ [file name: Multi-channel EEG recordings during a sustained-attention driving task (raw dataset)], and the pre-processed dataset^22^ [file name: Multi-channel EEG recordings during a sustained-attention driving task (pre-processed dataset)) to the publicly accessible repository of figshare.

## Technical Validation

### Behavioural validation

The EEG dataset was collected from 27 subjects with normal or corrected-to-normal vision. No subjects reported a history of psychiatric disorders, neurological disease or drug use disorders. All of the subjects were recruited university students and staff at the National Chiao Tung University, Taiwan. At the beginning of the experiment, each subject wore a suitable cap for recording the electrophysiological data and was given 5 to 10 minutes to read the experimental instructions and complete the participant information sheet (questionnaire).

The subjects’ facial videos and responses to the lane departure events were closely monitored. The experimenters visually observed the subjects’ facial features, such as eye movements (blink rate, blink duration, long closure rate, etc.), head pose and gaze direction via the surveillance video to determine whether the subject took his/her eyes off the road. Most importantly, the behavioural data (vehicle trajectory) objectively confirmed the estimated RTs during the experiment.

The RTs reflecting the participant’s promptness to respond to regular traffic events are considered an instantaneous measure of the level of fatigue and drowsiness. The RT to each lane-departure event (i.e., the time between the onset of the deviation and the onset of the response) was used as an objective behavioural measurement to characterise all EEG epochs. Three groups of epochs were defined: optimal-performance, suboptimal-performance, and poor-performance groups. Optimal-, suboptimal-, and poor-performance states might indicate that the participant performed the task with a low, intermediate, and high level of fatigue and drowsiness, respectively. For each subject, the RTs collected from the first 10 minutes of the experiment were used to construct a null distribution of optimal RTs.

**Fig. 4:**
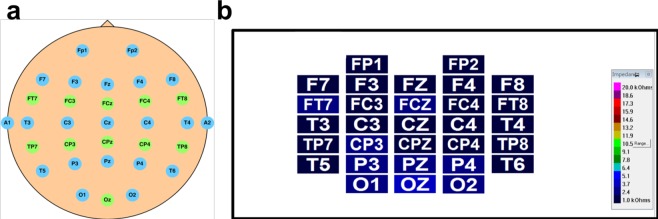
The layout of electrodes and impedance of electrodes in the EEG cap used in the experiments. (**a**) The blue electrodes use the international 10–20 system, and the green electrodes are additional electrodes on the cap. (**b**) The contact impedance between all of the electrodes and the skin was kept below 5 kΩ.

EEG signals were recorded using Ag/AgCl electrodes attached to a 32-channel Quik-Cap (Compumedical NeuroScan). Thirty electrodes were arranged according to a modified international 10–20 system, and two reference electrodes were placed on both mastoid bones, as shown in Fig. 4a. The skin under the reference electrodes was abraded using Nuprep (Weaver and Co., USA) and disinfected with a 70% isopropyl alcohol swab before calibration. Notably, as shown in Fig. 4b, the impedance of the electrodes was calibrated to be under 5 kΩ using NaCl-based conductive gel (Quik-Gel, Neuromedical Supplies®). EEG signals from the electro-cap were amplified using the Scan NuAmps Express system (Compumedics Ltd., VIC, Australia) and recorded at a sampling rate of 500 Hz with 16-bit quantisation.

### EEG validation

Consistent with previous data descriptors on practice reuse of EEG processing^23,24^, note that all EEG data including both raw and pre-processed versions, were saved in the figshare. In terms of the pre-processed dataset, all EEG data were saved after the pre-processing steps. The pre-processing steps included bandpass filters and artefact rejection. To be specific, raw EEG signals were subjected to 1-Hz high-pass and 50-Hz low-pass finite impulse response (FIR) filters. For artefact rejection, apparent eye blink contamination in the EEG signals was manually removed by visual inspection. Second, artefacts were removed by the Automatic Artifact Removal (AAR) plug-in for EEGLAB, which provided automatic correction of ocular and muscular artefacts in the EEG signals. The file names of the raw and pre-processed versions of the dataset are shown in Table 3.

Additionally, we shared this EEG dataset with our partner groups, including the University of California at San Diego (UCSD) and the DCS Corporation. Our findings are consistent with their results^25,26^, providing technical validation of this method for accurately estimating changes in driver arousal, fatigue, and vigilance levels by evaluating changes in behavioural and neurocognitive performance.

**Table 3:** The file names of the raw and pre-processed versions of the dataset.

Session	File Names	
	Raw Dataset	Pre-processed Dataset*
1	s01_051017m.set	s01_051017m.set.zip
2	s01_060227n.set	s01_060227n.set.zip
3	s01_060926_1n.set	s01_060926_1n.set.zip
4	s01_060926_2n.set	s01_060926_2n.set.zip
5	s01_060926_2n.set	s01_060926_2n.set.zip
6	s02_050921m.set	s02_050921m.set.zip
7	s02_051115m.set	s02_051115m.set.zip
8	s04_051130m.set	s04_051130m.set.zip
9	s05_051120m.set	s05_051120m.set.zip
10	s05_060308n.set	s05_060308n.set.zip
11	s05_061019m.set	s05_061019m.set.zip
12	s05_061101n.set	s05_061101n.set.zip
13	s06_051119m.set	s06_051119m.set.zip
14	s09_060313n.set	s09_060313n.set.zip
15	s09_060317n.set	s09_060317n.set.zip
16	s09_060720_1n.set	s09_060720_1n.set.zip
17	s11_060920_1n.set	s11_060920_1n.set.zip
18	s12_060710_1m.set	s12_060710_1m.set.zip
19	s12_060710_2m.set	s12_060710_2m.set.zip
20	s13_060213m.set	s13_060213m.set.zip
21	s13_060217m.set	s13_060217m.set.zip
22	s14_060319m.set	s14_060319m.set.zip
23	s14_060319n.set	s14_060319n.set.zip
24	s22_080513m.set	s22_080513m.set.zip
25	s22_090825n.set	s22_090825n.set.zip
26	s22_090922m.set	s22_090922m.set.zip
27	s22_091006m.set	s22_091006m.set.zip
28	s23_060711_1m.set	s23_060711_1m.set.zip
29	s31_061020m.set	s31_061020m.set.zip
30	s31_061103n.set	s31_061103n.set.zip
31	s35_070115m.set	s35_070115m.set.zip
32	s35_070322n.set	s35_070322n.set.zip
33	s40_070124n.set	s40_070124n.set.zip
34	s40_070131m.set	s40_070131m.set.zip
35	s41_061225n.set	s41_061225n.set.zip
36	s41_080520m.set	s41_080520m.set.zip
37	s41_080530n.set	s41_080530n.set.zip
38	s41_090813m.set	s41_090813m.set.zip
39	s41_091104n.set	s41_091104n.set.zip
40	s42_061229n.set	s42_061229n.set.zip
41	s42_070105n.set	s42_070105n.set.zip
42	s43_070202m.set	s43_070202m.set.zip
43	s43_070205n.set	s43_070205n.set.zip
44	s43_070208n.set	s43_070208n.set.zip
45	s44_070126m.set	s44_070126m.set.zip
46	s44_070205n.set	s44_070205n.set.zip
47	s44_070209m.set	s44_070209m.set.zip
48	s44_070325n.set	s44_070325n.set.zip
49	s45_070307n.set	s45_070307n.set.zip
50	s45_070321n.set	s45_070321n.set.zip
51	s48_080501n.set	s48_080501n.set.zip
52	s49_080522n.set	s49_080522n.set.zip
53	s49_080527n.set	s49_080527n.set.zip
54	s49_080602m.set	s49_080602m.set.zip
55	s50_080725n.set	s50_080725n.set.zip
56	s50_080731m.set	s50_080731m.set.zip
57	s52_081017n.set	s52_081017n.set.zip
58	s53_081018n.set	s53_081018n.set.zip
59	s53_090918n.set	s53_090918n.set.zip
60	s53_090925m.set	s53_090925m.set.zip
61	s54_081226m.set	s54_081226m.set.zip
62	s55_090930n.set	s55_090930n.set.zip
	Code-availability.zip**	
	Tutorial Data Analysis for Multi-channel EEG Recordings during a Sustained-attention Driving Task.pdf***	

*The pre-processing steps included bandpass filters and artefact rejection.

**EEG pre-processing and data analysis codes.

***Pre-processing and analysis guidelines for multi-channel EEG recordings during a sustained-attention driving task.

## Usage Notes

The raw experimental dataset^21^ and the pre-processed dataset^22^ can be downloaded from the publicly accessible repository of figshare. Any user interested in this dataset do not need to register with figshare to download two versions of the datasets, the raw and pre-processed versions, to the user’s personal computer. The raw and pre-processed versions of the dataset in the figshare projects are named “Multi-channel EEG recordings during a sustained-attention driving task (raw dataset)” and “Multi-channel EEG recordings during a sustained-attention driving task (pre-processed dataset)”, respectively.

The data can be analysed in EEGLAB, which is a MATLAB toolbox with an interactive graphical user interface (GUI). It includes multiple functions for processing continuous and event-related EEG using independent component analysis (ICA), time/frequency analysis and other methods, including artefact rejection under multiple operation systems. EEGLAB also provides extensive tutorials (https://sccn.ucsd.edu/wiki/EEGLAB_TUTORIAL_OUTLINE) to help researchers conduct data analyses. We recommend that researchers use EEGLAB with version 5.03 on Windows 7 or Linux.

A data analysis tutorial (named “Tutorial Data Analysis for Multi-channel EEG Recordings during a Sustained-attention Driving Task.pdf) and MATLAB codes (named “Code-availability.zip”) are provided as reference material for EEG pre-processing and data analysis during a sustained-attention driving task. These items can be accessed at our figshare webpage, ensuring that researchers can easily reuse the dataset.

Additionally, we provide some key notes regarding the data analysis.

1. Load the existing dataset. Select menu item ‘File’ and select the ‘Load existing dataset’ sub-menu item. Then, select the existing dataset (e.g., s01_051017m.set) from the sub-window pop up. If users use the pre-processed dataset, each file must first be decompressed, and then, the .set file should be selected.
2. Check the workspace in MATLAB. The ‘EEG’ variable contains the following information:
  srate: sampling rate
  EEG.chanlocs: the number of channels
  EEG.event: event type and latency
  data: EEG signals with channels multiply times
3. Extract data epochs and conduct further data analysis. To study the event-related EEG dynamics of continuously recorded data, we must extract the data epoch time of the events of interest (for example, the data epoch time of the onsets of one class of experimental stimuli) by selecting Tools > Extract Epochs. Additionally, removing a mean baseline value from each epoch is useful when there are baseline differences between data epochs (e.g., arising from low frequency drifts or artefacts). Additionally, EEGLAB contains several functions for plotting averages of dataset trials/epochs, selecting data epochs, comparing event-related brain potential (ERP) images, working with ICA components, decomposing time/frequency information and combining multiple datasets.
4. Considering the sample size calculation, if we consider a population size with 62 copies, a 95% confidence level, and a 5% margin of error, the minimum sample size should be 54 copies.

## ISA-Tab metadata file

Download metadata file

## Data Availability

Readers can access tutorial and codes in our raw and pre-processed datasets at the figshare.com. Of them, a 59-page tutorial named “Tutorial Data Analysis for Multi-channel EEG Recordings during a Sustained-attention Driving Task.pdf” is provided for researchers to pre-process and analyse multi-channel EEG data acquired during a sustained-attention driving task. Furthermore, MATLAB codes named “Code-availability.zip” for EEG pre-processing and data analysis can also be found.
